# Microbial Profile During Pericoronitis and Microbiota Shift After Treatment

**DOI:** 10.3389/fmicb.2020.01888

**Published:** 2020-08-05

**Authors:** Xiuling Huang, Hui Zheng, Jingang An, Shuo Chen, E Xiao, Yi Zhang

**Affiliations:** ^1^Third Clinical Division, Peking University School and Hospital of Stomatology, Beijing, China; ^2^National Engineering Laboratory for Digital and Material Technology of Stomatology, Beijing Key Laboratory of Digital Stomatology, yBeijing, China; ^3^Department of Orthodontics, School and Hospital of Stomatology, Fujian Medical University, Fuzhou, China; ^4^Department of Oral and Maxillofacial Surgery, Peking University School and Hospital of Stomatology, Beijing, China; ^5^The First People’s Hospital of Jinzhong, Jinzhong, China

**Keywords:** pericoronitis, 16S rRNA, microbiome, treatment, sequencing

## Abstract

The microflora of the distal pocket is considered as the major cause of pericoronitis. How the oral microflora changes during pericoronitis and whether different types of impacted third molar harbor the same microflora are still unknown. Saliva, subgingival plaque, and gingival plaque of mandibular third molars (M3Ms) were collected from twelve patients with acute pericoronitis. They were given local irrigation or local irrigation + antibiotics according to symptoms. Samples were harvested at the first visit with pericoronitis, 1 week after treatment, and 6 weeks after treatment. 16S rRNA gene polymerase chain reaction products were generated and sequenced after DNA isolation. Comparison of three sampling sites showed that, the subgingival plaque of M3Ms had most remarkable changes in symptomatic period, including a significant increase in microbial richness, and a convergent trend in microbial composition. After treatment, the subgingival microbiome was altered and largely returned to the state in asymptomatic period. In summary, the distal subgingival microbiota of M3M was most likely to be associated with the pathogenesis of pericoronitis. The post-treatment microbiota shift of M3M proved the effectiveness of treatment. The inclination type of impacted M3Ms and treatment method would also make a difference to the pericoronal microbiota.

## Introduction

Pericoronitis is defined as infection of the soft tissue surrounding the crown of a partially erupted tooth (Nitzan et al., 1985), which is most frequently the mandibular third molar (M3M). Its highest incidence is found in late adolescence and young adulthood, at the time of third molar eruption (Wade et al., 1991).

The microflora that develops in the distally located pseudo-pocket has been reported as the major cause of pericoronitis (Rajasuo et al., 1996; Sixou et al., 2003). Almost all previous studies on the microorganisms of pericoronitis chose the distally located pocket (or subgingival plaque) as the sampling site. Yet there is a doubt whether the microbiota at other oral sites would have certain changes during pericoronitis. Saliva contains bacteria shed from biofilms on every oral tissue (Costalonga and Herzberg, 2014), reflecting the overall level of oral bacteria. Gingival plaque of M3M might also affect the development of pericoronitis. Therefore, we also tried to investigate the microbiota changes at the above sites during pericoronitis.

It is known that the inclinations of M3Ms are classified as vertical, horizontal, mesioangular, or distoangular (Bishara and Andreasen, 1983). Blakey found that 80% of symptomatic M3Ms were vertical or distoangular (Blakey et al., 1996). The result was consistent with other studies (Renton et al., 2001). Since pericoronitis was more likely to occur in vertical impacted M3Ms, we wondered whether different inclination types of M3Ms harbored distinct microflora during pericoronitis.

Numerous studies have demonstrated that the microbial flora associated with pericoronitis is mixed and dominated by anaerobic bacteria (Leung et al., 1993; Rajasuo et al., 2007; Sencimen et al., 2014). They suggested a number of possible pathogens, including *Streptococcus*, *Staphylococcus*, and *Fusobacterium* species, etc. Yet more recent work has shown that, rather than some certain pathogens, the changes in microbial community may be the key factor contributing to the initiation, and/or persistence of many diseases including diabetes, asthma, and allergies, etc (Petersen and Round, 2014; Xiao et al., 2017). Limited by experimental method, most previous studies could only detect several periodontal pathogens instead of the entire microbiome. And they usually used healthy control instead of self-control.

The preferred treatment of pericoronitis is local treatment including debridement or irrigation. Antibiotic treatment is recommended to add when facial or systemic symptoms occur. Nonetheless, whether these two kinds of treatment would cause disparate changes to oral microbiota was still unknown.

Given the above, the study was aimed to investigate oral microbiota changes at three representative sites (saliva, subgingival, and gingival plaque of M3M) during pericoronitis and after treatment by sequencing of polymerase chain reaction (PCR)-amplified 16S rRNA gene, and attempted to find the association between microbial dysbiosis and the pathogenesis of pericoronitis. We also discussed whether the inclination type of impacted M3Ms and treatment method would have an impact on the oral microbiota in patients.

## Materials and Methods

### Patients

Adults patients without systemic diseases or pregnancy, suffering from M3M pericoronitis with obvious symptoms like pain or pus, etc. and without antibiotic treatment or local irrigation in the previous 3 months, were included in the study. Following treatment principles, patients with localized pain, and swelling involving pericoronal tissue would be given local irrigation with 0.02% potassium permanganate solution. When patients were exhibiting regional or systemic signs and symptoms (such as facial swelling and high fever, etc.), antimicrobial therapy would also be given (metronidazole, 0.4 *g* Tid po, 3–4 days). The study was approved by the Ethics Committee of Peking University School and Hospital of Stomatology (ID: PKUSSIRB-201525108). Written informed consent was obtained from all participants involved in the study. The research was conducted in full accordance with the World Medical Association Declaration of Helsinki.

General examinations included pain VAS, fever, facial swelling, and maximum mouth opening. Oral examinations included the gingival coverage on the crown, plaque index (PI; Ainamo and Bay, 1975), gingival index (GI; Loe and Silness, 1963), probing pocket depth (PD), and inclination type of M3M. PI, GI, and PD were acquired using a periodontal probe with 1.0 mm units. All measurements were made according to the standard. Radiographs were taken to exam the inclination type of M3M.

The time of sampling was at the presentation of patients with pericoronitis, 1 week after treatment, and 6 weeks after treatment. At the latter two time points, patients were free of symptoms and these samples were used as self-control. These three time points represented three periods patients underwent, namely symptomatic period, treatment period, and asymptomatic period. Each time for each patient, the samples included gingival, and subgingival plaque of M3M as well as saliva.

First, the patient bowed his head and put a large sterile test tube (NEST No. 602052, Wuxi, China) beneath his lower lip, and then opened mouth to make saliva naturally flow into the test tube to obtain a saliva sample. Second, isolated with cotton rolls, gingival plaque of M3M was collected and cleaned using a sterile cotton swab (Epicentre No. 4383, Madison, WI, United States). In the end, a sterile periodontal curette was gently inserted to the bottom of the distal pocket to collect subgingival plaque and the sample was transferred to a sterile cotton swab (Epicentre No. 4383, Madison, WI, United States). Then all three samples were kept in frozen tubes at the temperature of −20°C.

### Microbial DNA Extraction and Sequencing

Microbial genomic DNA was isolated using a QIAamp DNA micro Kit (Qiagen No. 51306, Hilden, Germany) in the microbiology laboratory at Peking University School of Stomatology. The V3–V4 hypervariable regions of the 16S rRNA gene were subjected to high-throughput sequencing by Beijing Auwigene Tech, Ltd (Beijing, China) using the Illumina Miseq PE300 sequencing platform (Illumina, Inc., CA, United States).

### 16S Data Processing

The raw sequencing data were analyzed using the pipeline tools of MOTHUR (Schloss et al., 2009) and QIIME (Caporaso et al., 2010). To retain only high-quality sequences for the downstream analysis, sequences that were less than 100 bp in length, contained one or more ambiguous base-calls (N), or had <90% quality scores >Q20 were filtered. After trimming and filtering, high-quality sequences were aligned to the Ribosomal Database Project (Cole et al., 2009) and were clustered into operational taxonomical units (OTU) using QIIME at a 97% similarity level. Before further analysis, singleton OTUs were removed.

### Statistical Analysis

Paired *t*-test was used to compare alpha diversities (the microbial diversity) between two different periods. Independent *t*-test was used to compare alpha diversities between two different groups. Differences in the relative abundances of taxa between different periods were analyzed using Wilcoxon matched-pairs signed rank test. Differences in weighted UniFrac distances (quantitative data displaying microbial community variation within groups) between local treatment group and combined treatment group were analyzed using the Mann-Whitney *U* test. *P* values < 0.05 were considered to indicate statistical significance.

## Results

### Clinical Information

Twelve adults aged 20∼34 years (three men and nine women, mean age 27.17 years) were included in the study (Table 1). The pain VAS was between 4 and 9 with a mean of 6. One patient (8.33%) had a fever and five patients (41.67%) had facial swelling. The maximum mouth opening was between 5 and 35 mm. 75% of the M3Ms were at most 30% visible (occlusal surface) and only 25% of the M3Ms were 50% visible (occlusal surface). 33.33% of the patients had a PI (plaque index) of 2 and 66.67% were 3. All the patients had a GI (gingival index) of 3. PD (probing pocket depth) of the distal pocket was between 5–7 mm. 41.67% of the M3Ms were horizontally impacted (HI) and 58.33% were vertically impacted (VI). According to the severity of symptoms, six patients were given local irrigation and the other six patients were given local irrigation plus antibiotic treatment.

**TABLE 1:** Patients’ information.

**No.**	**Gender**	**Age**	**M3M**	**Impaction**	**Pain VAS**	**Facial Swelling**	**Fever**	**MMO**	**Occlusal Eruption**	**PI**	**GI**	**Distal PD**	**Treatment**
1	F	20	Left	Vertical	6	N	N	30 mm	30%	2	3	7 mm	LT
2	F	23	Left	Vertical	4	Y	N	30 mm	30%	3	3	7 mm	LT + AT
3	F	23	Right	Vertical	5	N	N	33 mm	30%	2	3	7 mm	LT
4	F	27	Right	Vertical	5	Y	N	5 mm	30%	3	3	7 mm	LT + AT
5	F	25	Left	Vertical	5	N	N	30 mm	50%	3	3	6 mm	LT
6	F	26	Left	Vertical	8	N	Y	20 mm	30%	3	3	7 mm	LT + AT
7	F	28	Left	Horizontal	4	Y	N	35 mm	30%	3	3	5 mm	LT + AT
8	F	33	Right	Vertical	9	Y	N	8 mm	30%	3	3	7 mm	LT + AT
9	M	33	Left	Horizontal	6	N	N	20 mm	50%	2	3	6 mm	LT
10	F	27	Right	Horizontal	9	Y	N	20 mm	30%	3	3	5 mm	LT + AT
11	M	27	Left	Horizontal	5	N	N	30 mm	0	2	3	7 mm	LT
12	M	34	Right	Horizontal	6	N	N	35 mm	50%	3	3	5 mm	LT

*Abbreviation: F, female; M, male; M3M, mandibular third molar; N, none; Y, yes; MMO, maximum mouth opening; PI, plaque index; GI, gingival index; PD, pocket depth; LT, local treatment; and AT, antibiotic treatment.*

### Subgingival Plaque of M3M Changed the Most During Pericoronitis

**FIGURE 1:**
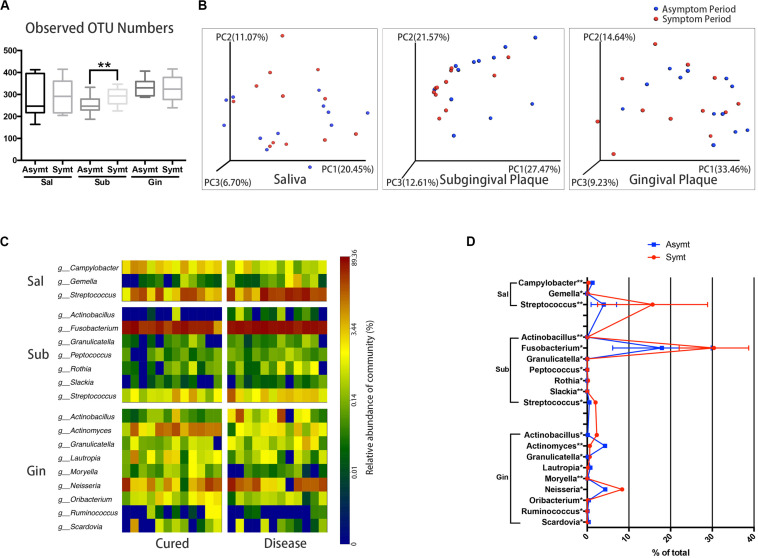
The oral microbiota changes from asymptomatic to symptomatic period at three sampling sites: saliva, Sal; subgingival plaque, Sub; and gingival plaque, Gin (*n* = 12). **(A)** Calculation of alpha diversity values (observed species) for comparison of the total microbial diversity of saliva, subgingival plaque, and gingival plaque between asymptomatic and symptomatic periods (paired *t*-test, *P < 0.05, and **P < 0.01). **(B)** The principal coordinate analysis charts based on weighted UniFrac distance: comparisons between asymptomatic and symptomatic periods at three sampling sites. **(C)** A taxa heat map of the relative abundances of bacteria that differed significantly between asymptomatic and symptomatic periods at three sampling sites, respectively (Wilcoxon matched-pairs signed rank test, *P* < 0.05). **(D)** The line chart showed the amount changes (relative abundance) of bacteria that differed significantly between asymptomatic and symptomatic periods at three sampling sites (Wilcoxon matched-pairs signed rank test, *P < 0.05, and **P < 0.01).

The differences in microbial diversity and microbial composition between asymptomatic and symptomatic periods were explored at different sampling sites. Among three sampling sites, only subgingival plaque of M3M had a statistically significant increase (*P* < 0.01) in OTU richness (microbial species richness) from the asymptomatic to symptomatic period (Figure 1A). The chart plotted based on principal coordinate analysis (PCoA) demonstrated that, subgingival plaque of M3Ms had an obvious tendency to cluster from the asymptomatic to symptomatic period, whereas gingival plaque of M3Ms and saliva showed a little tendency to disperse (Figure 1B). This suggested that, for different participants, their subgingival microbial composition of M3M became more similar when acute pericoronitis occurred. The taxa heat map and the line chart intuitively displayed the specific changes in bacterial composition of three sampling sites from the asymptomatic to symptomatic period (Figures 1C,D). The amount of *Fusobacterium* in subgingival plaque had increased greatly (*P* < 0.05). For gingival plaque, the *Neisseria* had increased significantly while the *Actinomyces* had decreased (*P* < 0.05). As for saliva, the *Streptococcus* had increased largely (*P* < 0.01).

### Horizontal and Vertical Impacted M3Ms Harbored Distinct Microbial Communities During Pericoronitis

**FIGURE 2:**
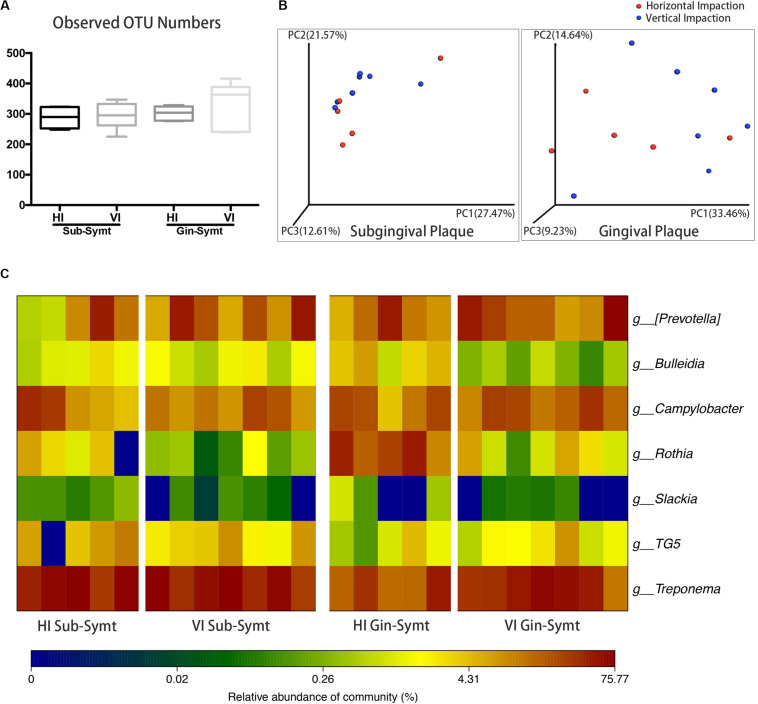
The comparisons of microbiota between the vertically impacted (VI, *n* = 7) M3Ms and horizontally impacted (HI, *n* = 5) M3Ms in symptomatic period: subgingival plaque, Sub; gingival plaque, Gin. **(A)** Alpha diversity values (observed species) of subgingival and gingival plaque were analyzed between HI and VI groups (independent *t*-test, *P* < 0.05). **(B)** The principal coordinate analysis charts based on weighted UniFrac distance: comparisons between HI and VI groups at two sampling sites. **(C)** A taxa heat map of the relative abundances of bacteria that differed significantly between the HI and VI groups in subgingival and gingival plaque (Mann-Whitney *U* test, *P* < 0.05).

The inclinations of M3Ms were classified as vertical, horizontal, mesioangular, or distoangular. According to this classification, our patients were divided into two groups: patients with VI M3Ms and patients with HI M3Ms. To investigate the differences of microbial community between two groups, we compared microbial diversity, and microbial composition in symptomatic period. There was no statistically significant difference in OTU richness either in subgingival or in gingival plaque between two groups (Figure 2A). However, both subgingival and gingival plaque of the two groups showed relatively significant separation in the chart plotted based on PCoA (Figure 2B), which meant the two groups tended to harbor different microbial communities during pericoronitis. A taxa heat map also confirmed the detailed differences in microbial composition between VI and HI groups (Figure 2C). For subgingival plaque, significant differences were found in relative abundance of *Rothia* (*P* < 0.05); for gingival plaque, such differences were found in *Rothia* (*P* < 0.01), *Treponema* (*P* < 0.05), and *Bulleidia* (*P* < 0.05).

### Microbial Communities of Subgingival Plaque Largely Reverted to the Asymptomatic-Period State After Treatment

**FIGURE 3:**
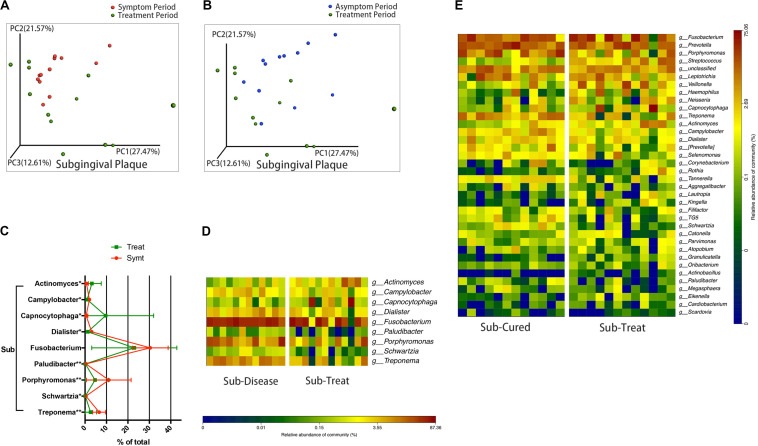
The subgingival microbiota changes from symptomatic to treatment period and the subgingival microbiota comparisons between asymptomatic and treatment periods (*n* = 12). **(A)** The principal coordinate analysis chart based on weighted UniFrac distance: comparison between symptomatic and treatment periods. **(B)** The principal coordinate analysis chart based on weighted UniFrac distance: comparison between asymptomatic and treatment periods. **(C)** The line chart showed the amount changes (relative abundance) of bacteria that differed significantly from symptomatic to treatment period in subgingival plaque (Wilcoxon matched-pairs signed rank test, *P < 0.05, and **P < 0.01). **(D)** A taxa heat map of the relative abundances of bacteria that differed significantly between symptomatic and treatment periods in subgingival plaque (Wilcoxon matched-pairs signed rank test, *P* < 0.05). **(E)** A taxa heat map of the bacterial composition of subgingival plaque in asymptomatic and treatment periods.

We compared microbial composition between different periods. After treatment, the convergent trend of subgingival microbial composition during pericoronitis was broken, namely, subgingival microbial communities seemed to return to a more diverse state in treatment period, just like that in the asymptomatic period (Figure 3A). The other chart plotted based on PCoA showed the distribution area of the subgingival plaque in treatment period were roughly coincident with that in asymptomatic period, which suggested subgingival microbial communities largely returned to the asymptomatic-period state after treatment (Figure 3B). A line chart and a taxa heat map gave details about the changes in microbial composition from pericoronitis to treatment (Figures 3C,D). After treatment, obligate anaerobes like *Porphyromonas* and *Treponema* (*P* < 0.01) decreased in subgingival plaque, and *Actinomyces* (*P* < 0.05) increased. The average relative abundance of *Fusobacterium* decreased with no significant difference. Another taxa heat map showed that the microbial composition of subgingival plaque during therapeutic and asymptomatic periods were similar (Figure 3E).

### Local Irrigation Combined With Systematic Antibiotic Made a Greater Microbiota Shift Than Local Irrigation Only

**FIGURE 4:**
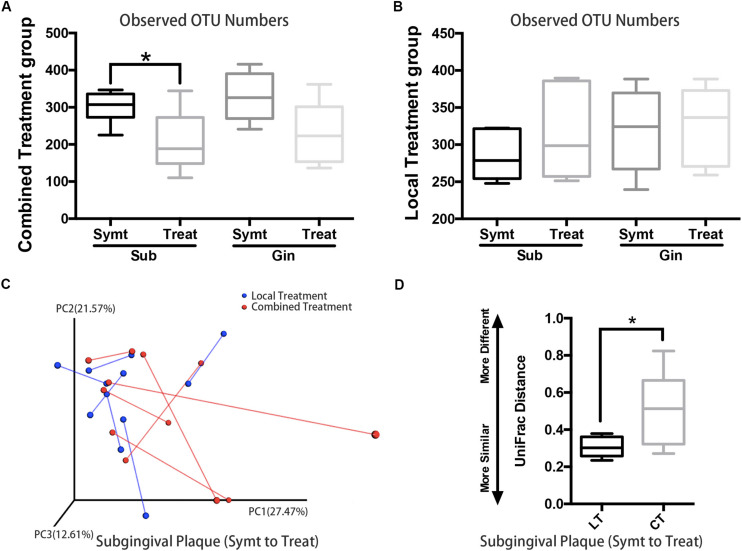
The comparison of microbiota changes brought by local (*n* = 6) and combined (*n* = 6) treatment: subgingival plaque, Sub; gingival plaque, Gin. **(A)** Alpha diversity values (observed species) were analyzed between symptomatic and treatment periods in combined treatment group (paired *t*-test, *P < 0.05). **(B)** Alpha diversity values (observed species) were analyzed between symptomatic and treatment periods in local treatment group (paired *t*-test, *P* < 0.05). **(C)** The principal coordinate analysis chart based on weighted UniFrac distance: comparison between local treatment and combined treatment groups. The UniFrac distance lines quantified the degree of the microbial composition variations of subgingival plaque from symptomatic to treatment period. **(D)** Comparison of UniFrac distance values of subgingival microflora from symptomatic to treatment period between local treatment (LT) and combined treatment (CT) groups (Mann-Whitney *U* test, *P < 0.05).

Patients with localized pain and swelling would be given local irrigation, while patients exhibiting regional or systemic signs and symptoms (such as facial swelling and high fever etc.) would be given combined treatment, which was local irrigation plus antibiotic therapy. We compared microbial diversities between pre- and post-treatment and found that, subgingival plaque of the combined treatment group had a statistically significant decrease (*P* < 0.05) in OTU richness (Figure 4A), while those of the local treatment group had no statistically significant change (Figure 4B). The PCoA chart confirmed the above result: the length of UniFrac distance lines represented the degree of the microbial composition variations from symptomatic to treatment period, and it showed that microbial composition of subgingival plaque in the combined treatment group changed more greatly than that in the local treatment group after treatment (Figure 4C). Statistical analysis results were also consistent with those of the chart: the UniFrac distance (quantitative data displaying microbial community variation within groups) between pre- and post-treatment of the combined treatment group was significantly more than that of the local treatment group (*P* < 0.05; Figure 4D).

## Discussion

Our results demonstrated that the distal subgingival plaque of M3M was most likely to be associated with the pathogenesis of acute pericoronitis. The result was consistent with previous studies (Rajasuo et al., 2007; Sencimen et al., 2014). More than clinical experience, we presented the evidence that microbial community changes of subgingival plaque were more remarkable than the other two susceptive sites. We noticed subgingival microbial composition of patients had a convergent shift when pericoronitis occurred. It suggested that subgingival plaque might have a certain pattern of microbial community composition that closely related to the occurrence of pericoronitis.

A number of studies have attempted to find the key microorganisms that associated with the pathogenesis of acute pericoronitis. Peltroche-Llacsahuanga et al. (2000) performed a study investigating infectious organisms causing pericoronitis by using phase-contrast microscopy and found that the *Streptococci milleri* group were most likely involved in the pathogenesis of acute pericoronitis. According to a study by Sixou et al. (2003) the most frequently detected microorganisms in pericoronitis microbiota were viridans group streptococci and those belonging to the genera *Actinomyces* and *Prevotella.* The above results were limited by traditional culture, since a significant fraction of species may not be detected owing to the stringent growth requirements. A recent study (Sencimen et al., 2014) based on quantitative real-time PCR found statistically significant higher numbers of *Tannerella forsythia* were detected in samples from pericoronitis patients. Although molecular studies of pericoronal bacteria using DNA probes and checkerboard DNA-DNA hybridization have provided additional insights, they are still limited to the detection of known bacterial species.

By using ribosomal RNA gene sequencing, we can identify known and previously unknown (and uncultivable) bacteria and therefore has the potential to provide a more complete description of microbial communities. In this study, we found the amount of *Fusobacterium* increased the most in subgingival plaque during pericoronitis. A previous study by Mansfield et al. (2012) also adopted the 16S rRNA gene sequencing, and showed that *Fusobacterium nucleatum* and *Dialister invisus* were in greater abundance in third molar sites exhibiting clinical symptoms. Their research differed from ours in that they used healthy controls rather than self-controls, so the slightly different results could be conceivable. As the most common species of *Fusobacterium*, *F. nucleatum* is a periodontal pathogen associated with a wide spectrum of human diseases, such as gastrointestinal disorders, cardiovascular disease and respiratory tract infections etc (Han, 2015). Therefore, it is likely that the increase of *Fusobacterium* is closely related to the pathogenesis of acute pericoronitis. Our study highlights the urgency of conducting further research on the role played by *Fusobacterium* in pericoronitis.

According to the inclination, M3Ms were classified as vertical, horizontal, mesioangular, or distoangular. A previous study (Hazza’a et al., 2009) showed that VI M3Ms were more common to be affected by pericoronitis than other types of M3Ms. To our knowledge, few previous studies had compared the microbial communities between different types of M3M. In this study, five HI M3Ms and seven VI M3Ms were collected and the results showed no significant difference in microbial richness, but obvious differences in microbial composition between two groups. In symptomatic period, the amount of *Treponema* was higher in VI M3M sites (in gingival plaque), and *Rothia* was higher in HI M3M sites (both in subgingival and gingival plaque). As one common species of the *Treponema* genus, *Treponema denticola* is known to be tightly related to periodontitis. The greater abundance of *Treponema* in gingival plaque of the VI M3Ms might explain the higher susceptibility of pericoronitis in VI M3Ms. Although the results are enlightening, studies of more samples should be conducted for further confirmation.

Through self-controlled design, the study attempted to minimize the bias due to individual variations and investigate whether the oral microbiota would be restored to the asymptomatic-period state after treatment. The results showed the convergent trend of subgingival microbial composition disappeared and it turned into a more diverse state after treatment. The taxa heat map showed the relative abundances of obligate anaerobes (such as *Fusobacterium, Porphyromonas*, and *Treponema*) reduced and those of facultative anaerobes (such as *Actinomyces*) increased, especially in subgingival plaque. Socransky et al. (1998) described the role of 5 main microbial complexes in periodontal diseases. Although our research was limited to genus, the above decreased obligate anaerobes contained three important species belonging to red or orange complexes, which were closely associated with periodontal diseases. Therefore, our results proved that treatment would cause a healthy shift in the oral microbiome, and this could also be confirmed by patients’ clinical manifestations. After treatment, the symptoms of pericoronitis diminished or disappeared, and microbial composition of subgingival plaque had largely returned to the asymptomatic-period state, which both proved the effectiveness of treatment. A literature survey revealed a lack of studies focusing on the post-treatment microbiome of the third molar pericoronitis. Therefore, the post-treatment results of the present study could not be compared with the results of a previous study.

Treatment of pericoronitis may require antibiotic therapy in addition to the procedures preserving regional hygiene such as operculectomy, debridement, and irrigation. Although it was generally agreed that local treatment was more efficient for pericoronitis than antimicrobial therapy (Moloney and Stassen, 2009), little research had been done to prove that. According to whether the symptoms were localized or diffuse, the patient would receive two kinds of treatment: local treatment (irrigation) or combined treatment (irrigation and antimicrobial therapy). The results showed combined treatment could make greater changes to the subgingival microbiome than local treatment, including more significant decrease of microbial enrichment and bigger changes in microbial composition. It could be concluded that, under the premise of local treatment, antibiotic treatment has the synergetic effect of treating pericoronitis.

In conclusion, our study investigated oral microbiota changes during pericoronitis and after treatment. The results demonstrated that the distal subgingival plaque of M3Ms was most likely to be associated with the pathogenesis of acute pericoronitis and the increase of *Fusobacterium* might play an important role therein. After treatment, microbial composition of subgingival plaque had largely returned to the state in asymptomatic period, which proved the effectiveness of treatment. Besides, we found that the inclination type of impacted M3Ms and treatment method would make a difference to the pericoronal microbiota.

## Data Availability Statement

The original contributions presented in the study are publicly available. This data can be found here: https://www.ncbi.nlm.nih.gov/bioproject, PRJNA606676.

## Ethics Statement

The studies involving human participants were reviewed and approved by the Ethics Committee of the Peking University School and Hospital of Stomatology. The patients/participants provided their written informed consent to participate in this study.

## Author Contributions

EX and YZ contributed to conception, design and data interpretation, and critically revised the manuscript. XH contributed to conception, design, data acquisition, analysis and interpretation, and drafted the manuscript. HZ contributed to data processing and analysis and critically revised the manuscript. JA contributed to analysis and interpretation and critically revised the manuscript. SC contributed to patient collection and sampling and critically revised the manuscript. All authors gave final approval and agreed to be accountable for all aspects of the work.

## Conflict of Interest

The authors declare that the research was conducted in the absence of any commercial or financial relationships that could be construed as a potential conflict of interest.
